# Psychological and sexual impact of human papillomavirus screening in women in Cameroon: a prospective cohort study

**DOI:** 10.1186/s12905-025-04083-6

**Published:** 2025-12-19

**Authors:** Jessica Sormani, Alida Moukam, Ania Wisniak, Virginie Yakam, Nicole C. Schmidt, Bruno Kenfack, Patrick Petignat

**Affiliations:** 1 Department of Paediatrics, Gynaecology and Obstetrics, Gynaecology Division, University Hospitals of Geneva, Avenue de Champel 47, Geneva, 1206 Switzerland; 2 Geneva School of Health Sciences, HES-SO, University of Applied Sciences and Arts Western Switzerland, Geneva, Switzerland; 3 Department of Obstetrics and Gynaecology, Dschang Annex Regional Hospital, Dschang, Cameroon; 4 Faculty of Medicine, Institute of Global Health, University of Geneva, Geneva, Switzerland; 5 Faculty of Social Science, Catholic University of Applied Science, Munich, Germany; 6 Department of Obstetrics and Gynaecology, Faculty of Medicine and Pharmaceutical Sciences, University of Dschang, Dschang, Cameroon; 7 Department of Paediatrics, Gynaecology and Obstetrics, Faculty of Medicine, University of Geneva, Geneva, Switzerland

**Keywords:** HPV screening, Sub-Saharan africa, Anxiety, Sexual dysfunction, Women’s health

## Abstract

**Background:**

Cervical cancer (CC) is a public health burden, particularly in low-and middle-income countries, but can be prevented by screening for Human Papillomavirus (HPV). Nevertheless, positive test results may have psychological and sexual impacts for women. This study aimed to assess the impact of HPV test results on anxiety and sexual dysfunction in Cameroonian women.

**Methods:**

This prospective cohort study conducted from 2020 to 2022 in Cameroon, included women previously enrolled in the 3T-Approach trial. Participants underwent a same-day consultation, organized as follows: HPV screening, triage of HPV-positive women and treatment by thermal ablation or Large Loop Excision of the Transformation Zone (LLETZ), if necessary. They completed anxiety (STAI) and sexual function (ASEX) questionnaires at 1, 6, and 12 months post-screening.

**Results:**

Among 273 women included in the study, 220 (80.6%) completed all time points. Upon inclusion, 24.5% were HPV-positive, of which 46.3% underwent treatment. Anxiety levels did not significantly differ by HPV status. Higher sexual dysfunction prevalence was observed in HPV-positive women at all intervals. Between 1 and 12 months HPV-positive women showed an increase in their mean sexual dysfunction score (+ 4.5, 95% CI: 0.5 to 8.4), which was not seen in HPV-negative women. This disparity persisted even after adjusting for socio-demographic factors.

**Conclusion:**

HPV test results did not significantly impact anxiety levels in women in Cameroon. Nevertheless, HPV-positive women reported higher sexual dysfunction at one-year post-screening compared to HPV-negative women. Further research should focus on investigating the factors affecting sexual dysfunction, in order to propose strategies to maintain women’s well-being without compromising screening rates.

**Trial registration:**

The protocol of the 3TApproach study was registered under ClinicalTrials.gov, (identifier NCT03757299) on 28, November, 2018.

**Supplementary Information:**

The online version contains supplementary material available at 10.1186/s12905-025-04083-6.

## Background

Cervical cancer (CC) is a global public health burden, which particularly affects low- and middle-income countries (LMICs) where 85% of CC cases occur [1], mainly caused by persistent infection with Human papillomavirus (HPV), is preventable by the means of HPV vaccination and screening [2].

Most women who are infected with HPV do not experience any symptoms and 80% of women eliminate the HPV infection on their own after 12 to 24 months [3, 4]. In the case of persistence, HPV infection may lead to precancerous cervical lesions, which can last several years before progressing to cancer [3, 4]. Thus, early detection of precancerous lesions allows adequate treatment and management, reducing CC cases and mortality.

In sub-Saharan Africa (SSA), CC is one of the leading causes of cancer deaths in women [1]. In this region, HPV prevalence is 24%, with a peak observed among women under the age of 25 [5]. In Cameroon, it is the second most common cancer with 2770 cases diagnosed in 2020 and the leading cause of cancer-related deaths among women [6]. Despite, its burden, only 6% of women aged 30 to 49 in Cameroon undergo CC screening [6], leaving the disease largely uncontrolled. Consequently, many cases go undetected or untreated due to limited access to adequate service.

The most effective way to prevent CC is through vaccination against HPV [7]. However, large-scale vaccination implementation is still several years away in the countries that need it the most [8]. In this context and with the public health objective to improve women’s access to healthcare and to reduce inequalities in the prevention and management of women’s health, the World Health Organization (WHO) published in 2021 guidelines for CC screening [2]. According to this recommendation for LMICs, the screen and treat method should be provided as adequate management to reduce CC and related mortality [2].

While HPV screening is unequivocally recommended, the process may trigger anxiety among women, leading to a sense of distress [9]. Women may face significant stigma and fear related to the infection and its potential impact on their health [10–13]. The disclosure of a positive HPV test result has mostly been demonstrated in high-income countries (HIC) as having adverse consequences on women’s mental well-being, leading to increased anxiety, sexual dysfunction, and sexual concerns [10, 14]. Furthermore, both receiving the HPV test result and undergoing a gynaecological examination are associated with elevated anxiety levels [15, 16]. Moreover, anxiety may persist for several months following screening [9]. After six months, however, levels of anxiety, distress and concern tend to diminish [17], and seem to return to standard levels by 12 months on average [18, 19]. Nevertheless, a few studies have reported that anxiety might persist beyond, potentially leading to low screening coverage and loss of women during follow-up if anxiety persists [10]. This highlights the importance of long-term follow-up of psychological outcomes and of implementing education approaches [11].

Despite the growing evidence on the psychological impact of HPV screening, there remains a dearth of research focusing on African populations, particularly regarding how screening and CC affect women’s mental and sexual well-being [20]. In SSA, psychosocial support services remain extremely limited [20, 21]. Access to specialized mental health care is low, and the region is underrepresented in organized psychosocial care for cancer patients [22, 23]. Although countries such as Nigeria and Ghana have established psycho-oncology societies, most countries lack dedicated infrastructure or professionals with psycho-oncology expertise to address the unique psychological needs of cancer patients [24–26]. In this context, we have already conducted a qualitative study in West Region of Cameroon to explore the cultural implications of HPV screening and its psychological impacts on women’s health [27]. This study provided valuable insights into the cultural and psychological factors influencing screening participation and healthcare delivery in the region. Building on these findings, the present study aims to assess the impact of HPV screening on anxiety levels and sexual dysfunction among women in the West Region of Cameroon, and how do these effects evolve over a 12-month period. Sexual dysfunction is defined as an alteration in sexual desire or physiological sexual response.

## Methods

### Setting and study design

This observational prospective cohort study was embedded within the 3T-Approach program, a CC screening trial in Dschang Annex Regional Hospital, Cameroon [28], from September 2020 to September 2022, launched by the University Hospital of Geneva, Switzerland.

The 3T-Approach program based on WHO recommendation to provide a sensitive context solution, offered a one-day consultation structured in three phases; (i) HPV DNA self-sampling (FLOQSwabs^®^; Copan, CA, USA) analysed via point-of-care assay (GeneXpert^®^ IV; Cepheid, Sunnyvale, CA, USA), (ii) triage of HPV-positive women through visual inspection with application of acetic acid (VIA) and Lugol’s iodine (VILI), and (iii) treatment by Thermal ablation (TA) or Large Loop Excision of the Transformation Zone (LLETZ) if triage results were positive. The final decision to treat the precancerous lesion was established by combining the results of the HPV self-sampling test and triage. The woman would undergo treatment only if she had positive results for both screen and triage procedures. Midwives advised women who had received treatment to refrain from sexual activity for 4 to 6 weeks post-treatment, which may have influenced the resumption of sexual activity at 1 month.

Women enrolled in the 3T-Approach program were consecutively recruited to participate in this ancillary study, following the same inclusion criteria as the original study: being aged between 30 and 49 years, no ongoing pregnancy, no history of hysterectomy or presence of CC symptoms and ability to comply with the study protocol.

### Procedures

At their first HPV screening consultation (baseline), and before undergoing the test, a medical anthropologist provided comprehensive information about the purpose and procedures of this ancillary study, including the administration of two questionnaires and the follow-up visits at 1, 6, and 12 months. Information about CC screening itself was delivered within the 3 T program by trained local midwives, following WHO recommendations.

Women who provided written consent at the first visit were enrolled in the study. Of those, only women who attended the one-month follow-up visit were included in the statistical analysis. Regardless of their HPV screening result at baseline, the anthropologist conducted structured questionnaire-based interviews at 1 month, 6 months and 12 months after screening. Two questionnaires, explained in the following section, were used to assess women’s anxiety and sexual dysfunction. The anthropologist was trained to maintain impartiality and avoid influencing the women’s responses to minimize response bias as much as possible.

In cases where women couldn’t attend in person due to personal reasons or COVID-related restrictions, phone interviews were conducted to complete the questionnaires. The anthropologist made reminder phone calls one week, three days, and one day before each scheduled visit. In addition, transportation costs were reimbursed based on a flat-rate system, calculated according to the distance between the participant’s residence and the screening center. The last 12-month follow-up visit took place in September 2022. All collected data were transcribed into a database using the SecuTrial^®^ software (Berlin, Germany).

### Measures

The primary outcomes were anxiety, which was assessed using the Short-Form State Anxiety Inventory-6 (STAI) questionnaire a 4-point Likert scale, with the total score ranging from 20 to 80 [29]. Scores below 35 indicate very low anxiety, scores ranging from 36 to 45 indicate low anxiety, from 46 to 55 medium anxiety, from 56 to 65 high anxiety, and scores above 65 indicate very high anxiety. In the literature, the mean score and standard deviation (SD) for adult women from HIC is 35 (± 10) [30]. Therefore, we considered scores exceeding 35 as indicative of elevated anxiety compared to general population. To ensure accuracy, we use a validated version of the STAI in French for Francophone women [31].

The Arizona Sexual Experience Scale (ASEX) questionnaire is a 6-point Likert scale evaluating sexual function. For each of the five questions, a score of 1 indicates sexual hyperfunction, while a score of 6 signifies sexual hypofunction, with the final score ranging between 5 and 30. The ASEX is specifically designed to assess five core components of sexual function identified in the literature as central to sexual disorders: sexual drive, arousal, vaginal lubrication, ease of achieving orgasm, and satisfaction with orgasm. Its low level of intrusiveness allows for repeated use while minimizing embarrassment for respondents [32]. A score of 18 or above is indicative of sexual dysfunction. For this tool as well, the validated French version of the ASEX was used for Francophone women [33].

The ASEX assumes recent sexual activity (i.e., during the past week) administering it to women who had not resumed sexual intercourse would have yielded unreliable or uninterpretable results. Therefore, to ensure temporal relevance between sexual experience and the HPV screening procedure, women who had not resumed sexual activity after HPV screening did not complete the ASEX at this time point. However, they were able to fill the ASEX at later time points if they had resumed sexual activity. For those who remained abstinent, the reasons were explored through open-ended questions. Women who were not sexually active could still complete the STAI. Interviews were conducted in French or English depending on each participant’s spoken language.

Patient’s sociodemographic characteristics and medical history were collected at baseline by trained midwifes through the 3T-Approach study.

### Statistical analysis

The sample size was calculated to detect a minimum decline of 5 points on the STAI mean score between 1 month and 12 months in both HPV-positive and HPV- groups in line with findings from a Finnish study [30]. A change of approximately 6.7 points on the STAI has previously been reported as clinically meaningful (Drolet et al., 2012); therefore, our choice of 5 points represents a conservative but clinically relevant threshold. The calculation assumed a power of 80% and for a two-sided paired t test and a significant level 0.05. For this purpose, we assumed a standard deviation of 10 [30] and, to ensure a robust estimate, conservatively considered no correlation between the STAI scores at 1 month and 12 months.

In the actual analysis, however, we assessed individual changes in STAI and ASEX scores for participants with data at both time points, thereby accounting any potential correlation between the two measurements.

Based on an expected prevalence of 30% HPV-positive among screened women and anticipating a 30% loss to follow-up, the required sample size was determined to be 270 women (203 HPV- and 67 HPV-positive). Recruitment for the study continued until the calculated sample size was reached for the first questionnaire one month after screening.

Quantitative variables were presented as means and standard deviations or medians and interquartile ranges depending on the distribution, while categorical variables were presented as percentages, unless stated otherwise. The mean decline of the STAI and ASEX scores, was calculated between 1 month and 6 months, as well as 1 month and 12 months after receiving the HPV result. Analyses of score changes over time included only participants with valid data at both time points, ensuring intra-individual comparisons without imputation. Additionally, the mean decline between time periods was compared between HPV status sub-groups (HPV-negative/HPV-positive). Categorical variables were analysed using Pearson’s chi-square or Fisher’s test, while continuous and ordinal variables by Student’s test for unpaired comparisons and the paired t test for paired comparisons, after verification of normal distribution. Additionally, multivariate linear regression models were conducted to adjust for socio-demographic factors associated with change in sexual dysfunction scores from 1 month. We used a two-sided level of significance of 0.05. The analyses were carried out using the software package STATA^®^ 16 (Stata, College Station, TX, USA).

## Results

### Epidemiological characteristics

**Fig. 1:**
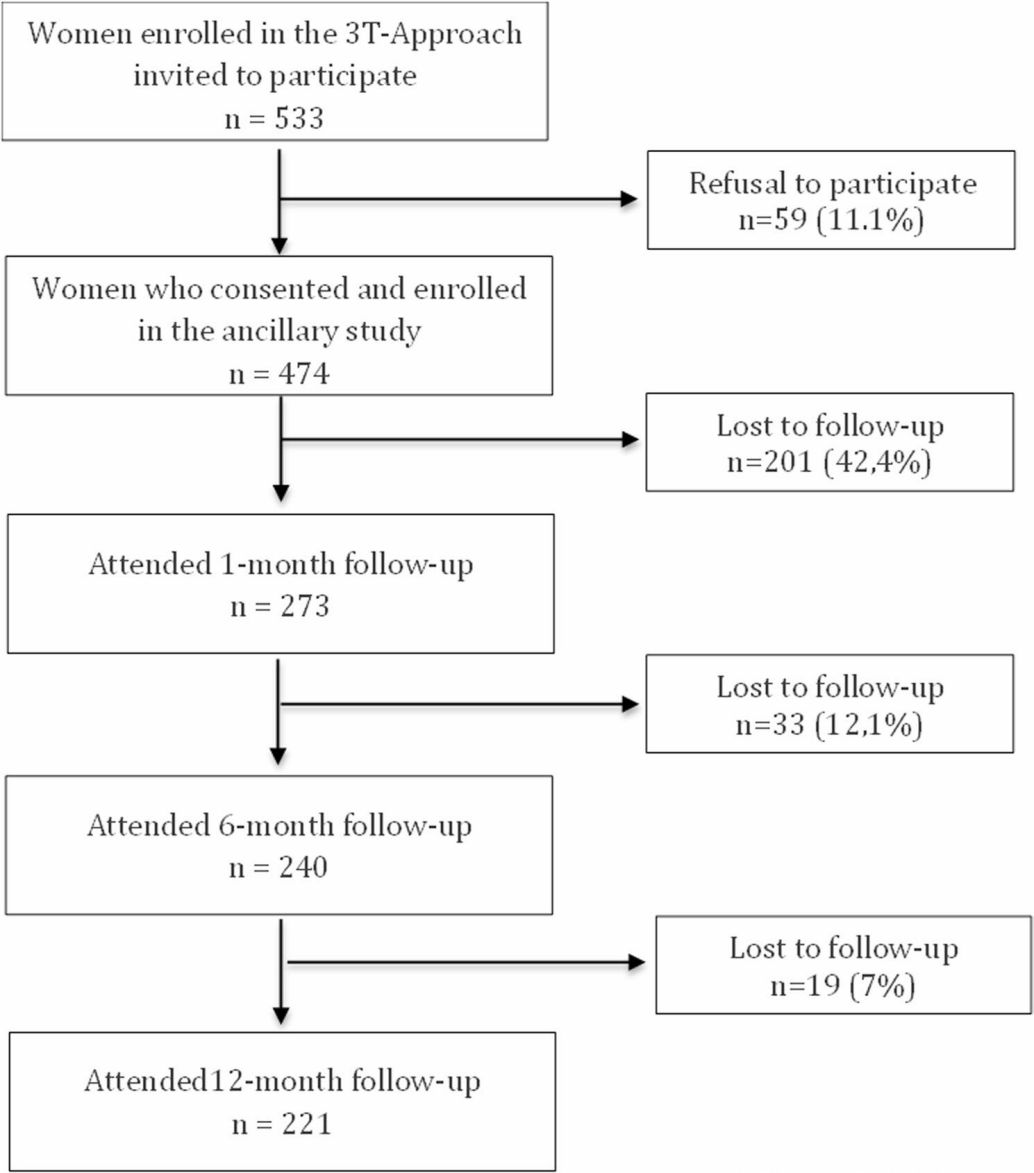
Flow chart of participant inclusion and follow-up

In the 3T-Approach program, a total of 533 women were invited during the recruitment period of this ancillary study. Among them, 474 consented to participate and were included in the study. However, only 273 (57.6%) of the included women attended the first visit at one month post-screening. Among the 273 women, 240 completed the STAI at 6 months, and 221 at 12 months. However, one woman who completed the STAI at 12 months had not completed it at 6 months. Consequently, 220 women (80.6%) completed the STAI at all three study visits thus contributing to the full longitudinal analysis of the primary outcome (see Fig. 1).

The median age was 39 years (Interquartile range (IQR): 44 − 34), and over 80% were either in a relationship or married. No significant differences in sociodemographic and medical history were observed between HPV-negative women and HPV-positive women, except for the age at first delivery, which was lower in HPV-positive women (*p* = 0.041). There were no significant differences within the HPV status concerning their HPV screening history, with approximately 14% having undergone prior HPV screening. During the initial baseline visit, the prevalence of HPV was 24.5%, and among women who tested positive for HPV, 31 (46.3%) had received treatment. All were treated by thermal ablation, except for one woman who had a cancer. About 6.2% of the women self-reported being HIV-positive. (see Table 1)

**Table 1:** Baseline sociodemographic, and clinical characteristics according to HPV status

Variable	HPV-Negative (*n* = 206) *N* (%)	HPV-Positive (*n* = 67) *N* (%)	*p* value	Total *N* (%)
Marital status			0.163	
Single/divorced/widowed	31 (15.1)	15 (22.4)		46 (16.8)
Married/In a relationship	175 (84.9)	52 (77.6)		227 (83.2)
Education			0.134	
Unschooled/Primary education	50 (24.3)	21 (31.3)		71 (26.1)
Secondary	118 (57.3)	29 (42.3)		147 (53.8)
Tertiary education	38 (18.5)	17 (25.4)		55 (20.1)
Employment status			0.633	
Student/Housewife/unemployed	33 (16.0)	12 (17.9)		45 (16.5)
Employee/independent	160 (77.7)	53 (79.1)		213 (78.0)
Work with responsibility	12 (5.8)	2 (3.0)		14 (5.1)
Missing value	1(0.5)	0 (0.0)		1 (0.4)
Age (y), median (IQR)	39 (34 − 44)	39 (33 − 44)		39 (34 − 44)
Age at first delivery (y), mean ± sd	21.3 (4.8)	19.8 (6.5)	0.041	21.0 (5.3)
Age at first intercourse (y), mean ± sd	18.4 (3.0)	18.3 (3.1)	0.729	18.4 (3.0)
Pregnancy			0.416	
Nulliparous	4 (1.9)	3 (4.5)		7 (2.6)
1–5	107 (51.9)	37 (55.2)		144 (52.7)
> 5	95 (46.1)	27 (40.3)		122 (44.7)
Parity			0.115	
0	6 (2.9)	5 (7.5)		11 (4.0)
1–5	146 (70.9)	40 (59.7)		186 (68.1)
> 5	54 (26.2)	22 (32.8)		76 (27.9)
VIA/VILI results				
Negative	NA	36 (53.7)		36 (53.7)
Positive	NA	31 (46.3)		31 (46.3)
Previous HPV test			0.533	
No	177 (85.8)	58 (86.6)		235 (86.1)
Yes	29 (14.1)	9 (13.4)		38 (13.9)
HIV status (self-reported)			0.092	
Negative	191 (92.7)	57 (85.1)		248 (90.8)
Positive	10 (4.9)	7 (10.4)		17 (6.2)
Missing value	5 (2.4)	3 (4.5)		8 (2.9)

*HIV* Human Immunodeficiency virus, *HPV* human papillomavirus, *VIA/VILI* visual inspection with acid acetic/visual inspection with lugol iodine, *y* years

### Anxiety

**Fig. 2:**
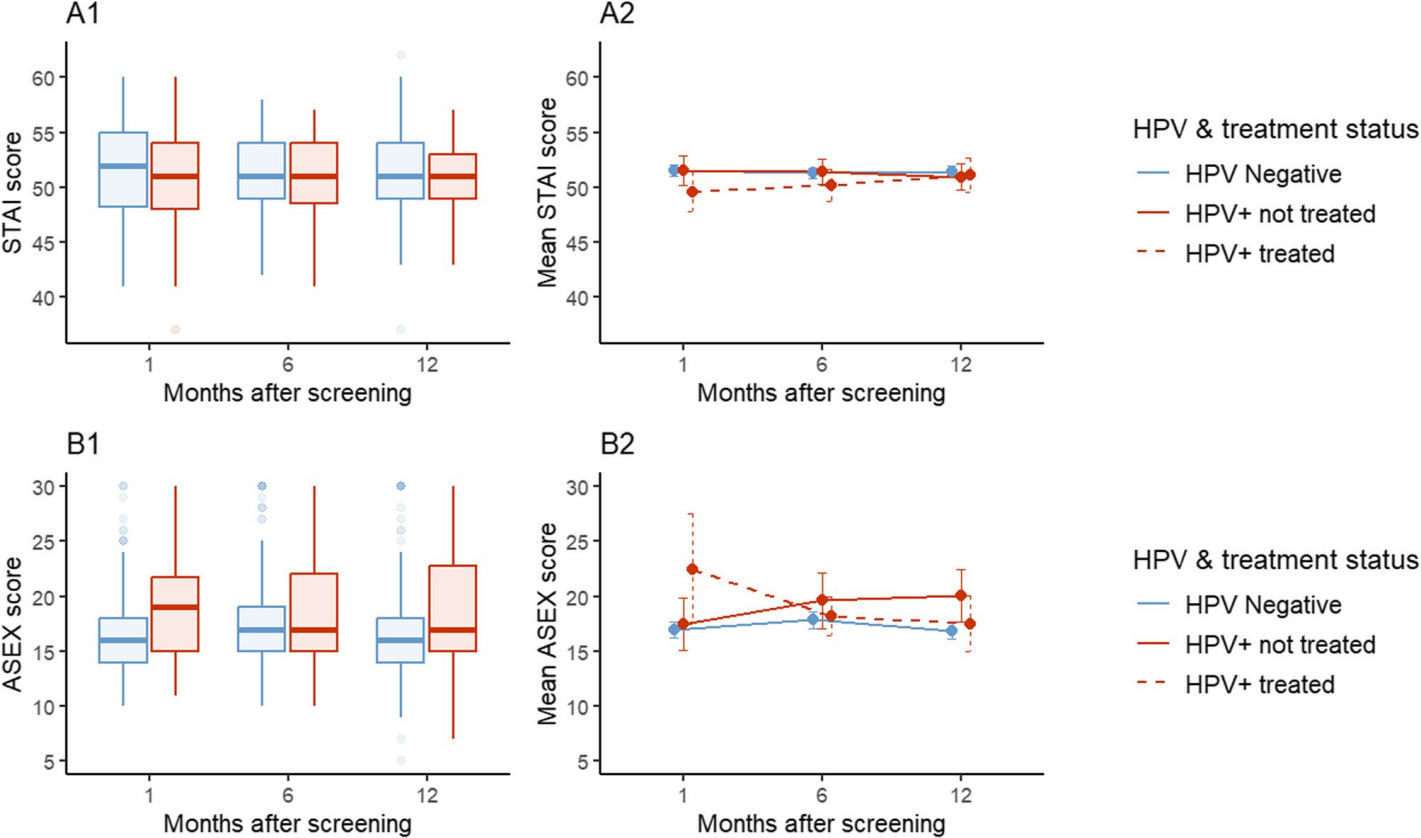
Temporal distribution and evolution of STAI and ASEX scores at 1, 6, 12 months after screening, **A1**. Distribution of STAI score by HPV status. **A2** Change in mean STAI score by HPV and treatment status with 95% CI. **B1** Distribution of ASEX score by HPV status. **B2** Evolution in mean ASEX Score by HPV and treatment status with 95% CI. For all boxplots, horizontal lines denote medians; boxes extend from the 25th to the 75th percentile; whiskers indicate adjacent values (within 1.5 IQR); and dots represent outliers. Note: STAI score: < 35 (very low); 36–45 (low); 46–55 (medium); 56–65 (high); >65 (very high). ASEX: <18 non-sexual dysfunction; ≥ 18 sexual dysfunction

Regardless of their HPV results, the mean anxiety scores ranged between 50.6 (sd 4.5) and 51.5 (sd 4.0) in all three periods, corresponding to a level of ‘medium anxiety’ (see Table 2; Fig. 2). Difference in the mean anxiety scores between HPV status was not statistically significant at either (see Table 2). High anxiety was reported by 15.1% of HPV-negative and 16.4% of HPV-positive at 1 month, decreasing at 6 months (9.6% vs. 4.8%), and increasing slightly again at 12 months (13.3% vs. 10.9%).

**Table 2:** Progression of mean STAI score 1, 6 and 12 months post-screening

Variable	1 month	6 months	12 months	Δ 6 to 1 mo (95% CI)	Δ 12 to 1 mo (95% CI)
HPV-negative (*n*)	206	177	166	−0.4 (− 1.1–0.3)	0.3 (− 1.0–0.4)
mean (sd)	51.5 (4.0)	51.3 (3.2)	51.4 (3.6)		
HPV-positive (*n*)	67	63	55	0.5 (− 0.8–1.8)	0.5 (− 0.9–1.9)
mean (sd)	50.6 (4.5)	50.9 (3.4)	51.0 (3.5)		
Difference HPV status (95% CI)	0.9 (− 2.1–−0.3)	0.5 (− 0.5–1.4)	0.4 (− 0.7–1.5)	−0.9 (− 2.4–0.6)	−0.8 (− 2.3–0.7)
*P* value	0.149	0.354	0.466	0.244	0.328

*HPV* human papillomavirus, STAI score < 35 (very low); 36–45 (low); 46–55 (medium); 56–65 (high); >65 (very high)

Similar trends were observed in the change in prevalence of high anxiety between 1, 6 and 12 months in both HPV status, with a decrease in the proportion of high anxiety at 6 months, and an increase at 12 months. However, when analysing the evolution of the mean anxiety score between periods, no statistically significant differences were observed between HPV status from 1 to 6 months (−0.9, 95% CI: −2.4 to 0.6, *p* = 0.244), and from 1 to 12 months (−0.8, 95% CI: −2.3 to 0.8, *p* = 0.328) (see Fig. 2, and Table 2). No difference in the evolution was observed between HPV-positive and HPV-negative women, nor between treated and untreated HPV-positive women.

### Sexual dysfunction

At one-month post-screening, 63.1% (*n* = 130) of HPV-negative women had resumed sexual activity since their screening visit. This proportion decreased to 44.4% (*n* = 16) in non-treated HPV-positive women and 19.4% (*n* = 6) in treated HPV-positive women. The primary reasons for the absence of sexual activity one month after screening varied across groups. Among abstinent HPV-positive untreated women (*n* = 20), the absence of a partner (i.e. no partner, geographical distance or being a widow) or relationship conflicts were reported by 16 women (80.0%). This percentage was slightly lower among abstinent HPV-negative women (69.7%). Additionally, 14 abstinent HPV-negative women (18.4%) reported other factors such as pain, illness, accidents, or periodic abstinence. For abstinent HPV-positive treated women, the most common reason was being in the post-intervention period (96%). Across all women included in the study, 7 (6.4%) reported living in polygamous households. Over time, an increase in sexual activity was observed in all subgroups, reaching nearly 90% in HPV-negative women, 90% in HPV-positive women untreated, and 78.3% in HPV-positive treated women after one year.

Sexual dysfunction was more prevalent among HPV-positive women compared to HPV-negative at all time points. At 1 month, sexual dysfunction was reported by 30.0% of HPV-negative women (39/130) and 54.6% of HPV-positive women (12/22). Prevalence increased at 6 months to 40.1% (59/147) and 48.8% (20/41) respectively, and remained high at 12 months, with 31.5% (47/149) and 45.7% (21/46) reporting sexual dysfunction (see Table 3). At this timepoint, HPV-positive women exhibited significantly higher mean sexual dysfunction scores compared to HPV-negative women (*p* = 0.028; see Table 3).

**Table 3:** Progression of mean ASEX score 1, 6 and 12 months post-screening

Variable	1 month	6 months	12 months	Δ 6 to 1 mo (95% CI)	Δ 12 to 1 mo (95% CI)
HPV-negative (*n*)	130	147	149	106	99
mean (sd)	17.0 (4.2)	17.9 (4.6)	16.9 (4.8)	1.6 (0.6–2.6)	0.0 (− 0.9–0.9)
HPV-positive (*n*)	22	41	46	21	19
Mean (sd)	18.9 (5.0)	19.0 (5.0)	19.0 (5.8)	1.1 (− 2.5–4.6)	4.5 (0.5–8.4)
Difference HPV status (95% CI)	−1.9 (− 4.2–0.4)	−1.1 (− 2.9–0.6)	−2.1 (− 4.0–−0.2)	0.5 (− 3.1–4.2)	−4.5 (− 8.5–−0.4)
*P* value	0.101	0.196	0.028	0.770	0.031

Concerning the evolution in the mean ASEX score over time, there was a statistically significant increase from 1 to 6 months in HPV-negative women (+ 1.6, 95% CI: 0.6 to 2.6) but not from 1 to 12 months (Fig. 2; Table 3). In HPV-positive women, the increase was statistically significant only from 1 to 12 months (+ 4.5, 95% CI: 0.5 to 8.4) (see Fig. 2; Table 3). The increase in ASEX score was larger in HPV-positive than in HPV-negative from 1 to 12 months (+ 4.5, *p* = 0.031) but not from 1 to 6 months (−0.5, *p* = 0.770). The change in mean ASEX score over time by treatment status is presented in Fig. 2, panel B2. These differences in increase between HPV-positive and HPV-negative were not modified when adjusted for all socio-demographic factors (see Additional file 1).

## Discussion

Women irrespective of their HPV status, exhibited minimal fluctuation in their anxiety levels during the first year following their screening test, with a decrease in the prevalence of high anxiety with time in both groups. This trend aligns with results found in previous studies in Europe and Canada [14, 18, 34]. Results from a qualitative study, conducted in HPV primary screening sites in England, revealed that HPV-positive women with elevated anxiety expressed concerns about the 12-month interval for routine follow-up. Their apprehensions were rooted in the possibility of developing CC during this waiting period [35].

Overall, women in our study population displayed an average anxiety score above the upper threshold expected in the general population based on Western norms (mean score exceeding 50 compared to the expected range of 34–36) [30]. Therefore, these findings should be contextualized within the context of LMICs where anxiety is more prevalent [36]. Furthermore, results should be compared especially among those living in conflict zones or who have experienced conflict in the previous 10 years, as 22.1% of individuals in post-conflict areas present mental disorder, including anxiety [37]. The study site, situated in the region of Dschang, is in close proximity to the ongoing Anglophone crisis, which might explain women’s higher anxiety score, although this warrants further investigation in subsequent studies [38]. Other systematic reviews and meta-analyses predominantly employing the STAI as a measurement tool, have also highlighted a higher prevalence of self-reported anxiety symptoms among women in the perinatal period in LMICs compared to HIC, possibly influenced by cultural factors [39, 40] or linguistic factors that could affect the validation of screening tools [36]. These findings highlight the importance of adopting differentiated approaches in mental health management according to cultural context underscoring the need for thorough evaluation in future studies.

HPV-positive women reported a higher level of sexual dysfunction in the year following screening compared to HPV-negative women. The higher sexual dysfunction among HPV-positive women is consistent with existing literature in the Global North and in Asia, which suggests that receiving a positive HPV result may lead to psychological distress and adversely affect sexual well-being [10, 12, 41–43]. A systematic review similarly revealed that women who were tested positive for HPV showed significantly heightened concerns about their sexual health at 6-months compared to those who tested negative for HPV [42].

In the literature, disruptions in sexual function and well-being among women have been associated with fear of developing cancer, experiences of stigma, concern about a partner’s infidelity, awareness of the implications of an HPV infection, and the subsequent medical care required [12, 13, 43, 44]. For example, in England, women have reported that being aware of their HPV-positive diagnosis complicates the prospect of forming future sexual relationships [13]. Knowledge of one’s positive HPV status often triggers feelings of guilt and apprehension about disclosure, leading some women to choose to conceal their HPV infection from partners and refrain sharing their test results with others [13, 44].

In relationships, HPV infection may alter dynamics, introducing uncertainty, or misunderstandings about fidelity [12]. Some women fear that a partner might feel betrayed or view them negatively, heightening stress and potentially contributing to a cycle of guilt and apprehension [12]. Moreover, the fear of infecting or reinfecting one’s partner may intensify in case of delayed follow-up or uncertainty about the clearance of the virus. These concerns may be exacerbated by limited access to information, particularly in LMICs, resulting in misconceptions about HPV transmission, risks, and follow-up. These misconceptions may increase sexual dysfunction.

Bennett et al. reported that transmission of HPV and its potential impact on a sexual partner are key topics on which women seek more information, noting psychosexual distress may be exacerbated by having to wait for confirmation that the infection has cleared [44]. These findings underscore the importance of addressing common questions and concerns about infectivity and transmission in informational materials provided to women who test HPV positive, as highlighted in other studies [45].

Furthermore, the study’s results shed light on the possible influence of chronic care processes comprising frequent clinical visits and ongoing monitoring, on sexual dysfunction overtime. Our results showed on gradual increase on sexual dysfunction in HPV-positive women between 1 month, 6 months and 12 months following screening. Repeated follow-ups can heighten anxiety or alter how women perceive their bodies and sexuality over time.

Our findings reveal a tendency of an increase sexual dysfunction on HPV-positive untreated women compared to those having received treatment. However, the limited number of participants who received treatment for precancerous lesions made it difficult to draw definite conclusions. In the literature, one study conducted in Turkey found that HPV-positive women who had not yet received any related treatment experienced significantly greater reductions in desire, arousal, and lubrication, along with more difficulty reaching orgasm, compared to a control group [43]. In contrast, Hellsten et al. (2008) observed no difference in sexual functioning between women who underwent LEEP and those who did not.

Training healthcare providers is crucial to ensure they effectively communicate the implications of HPV results, including sensitive topics such as the sexual and relational consequences of a positive diagnosis [10, 18, 35], while also providing psychological support to address concerns about relationships, stigma, and potential partner rejection. By integrating tailored information, counselling, and education into follow-up, it may be possible to reduce sexual dysfunction and improve overall well-being throughout the screening process among women receiving their first HPV-positive result [11].

One limitation of the study is the potential selection bias, as approximately 40% of eligible women did not attend the visit at 1 month, which may have reduced the representativity of the sample. Additionally, a response bias is possible due to the sensitive nature of the topic and the potential influence of stigma. To address this, a trained medical anthropologist conducted interviews to minimize bias, although she was not blinded to participants’ HPV results, a potential source of observer bias. Of note, it was not feasible to collect anonymous responses via paper forms due to participants’ low literacy levels. Other limitations include the absence of data regarding anxiety levels and sexual dysfunction of women within the study population prior to CC screening, as well as data from the general population in this context, which constitutes a major limitation. Additionally, the small sample size of HPV-positive women who completed the ASEX limits the generalizability of the findings. Furthermore, among women who remained sexually inactive, the lack of a formal measurement tool such as ASEX prevented us from directly assessing changes in sexual interest. However, qualitative data collected through open-ended questions did not identify loss of desire as a reason for abstinence, which may partially mitigate this limitation. One further limitation is the lack of formal validation of the STAI and ASEX in the Cameroonian population. The STAI has been used in other LMICs, which provides a reasonable basis for its application in this study [46]. For the ASEX, no validation has been conducted in this context, which we acknowledge as a limitation. The COVID-19 pandemic may have influenced anxiety and participation in screening [47–49]. Although all participants were exposed to the same contextual factors, likely reducing differential bias between groups, no studies to date in our setting have assessed whether pandemic-related anxiety affected HPV-positive and HPV-negative women differently. A qualitative study from Zimbabwe reported a decline in screening uptake due to fear of infection, but did not differentiate screening behaviour by HPV status [48], thereby limiting the possibility of direct comparison with our findings.

A notable strength lies in a majority of women being able to attend the third consultation regardless of their screening results, thereby enabling a one-year follow-up post-screening.

## Conclusion

These findings emphasize the potential impact of HPV screening results on women’s sexual dysfunction, particularly within the context of a chronic care process. They underscore the need for healthcare providers to implement comprehensive care approaches that include psychological counselling, tailored education about HPV transmission and follow-up, and open communication to address patients’ concerns about relationships, stigma, and partner dynamics. Developing targeted support programs and integrating psychosocial support into follow-up care could help mitigate the burden of sexual dysfunction associated with HPV screening. Further research should investigate factors influencing sexual dysfunction in screened women to develop strategies that support women’s well-being. Additionally, longitudinal studies following up women from this study could provide valuable insights into the long-term psychological and sexual health impacts of HPV screening.

## Supplementary Information

Supplementary Material 1

## Data Availability

The datasets analysed during the current study are available from the corresponding author on reasonable request.
