# Supplementary material for: Psychological and sexual impact of human papillomavirus screening in women in Cameroon: a prospective cohort study
Source: BMC Womens Health. 2025 Dec 19;25:602. doi: 10.1186/s12905-025-04083-6 (PMC12717717; doi:10.1186/s12905-025-04083-6)
Supplement: Supplementary file 1 — Supplementary Material 1 [file 12905_2025_4083_MOESM1_ESM.docx]

**Addition file 1:**

**Table 4**: Association of socio-demographic factors with change in sexual dysfunction from 1 month

|  | **Change from 1 to 6 months** | | **Change from 1 to 12 months** | |
| --- | --- | --- | --- | --- |
| **Sociodemographic variables** | **Adjusted**  **mean difference (CI 95%)** | **pvalue** | **Adjusted**  **mean difference (CI 95%)** | **pvalue** |
| HPV status |  |  |  |  |
| Negative | Ref |  | Ref |  |
| Positive | -1.07 (-3.91; 1.77) | 0.457 | 3.88 (0.92; 6.85) | **0.011** |
| Civil status |  |  |  |  |
| Married/in relationship | Ref |  | Ref |  |
| Single/divorced/widowed | 2.16 (-2.09; 6.41) | 0.316 | 1.31 (-2.90; 5.51) | 0.539 |
| Education |  |  |  |  |
| Unschooled/primary education | Ref | *0.459** | Ref | *0.650** |
| Secondary education | -1.20 (-4.00–1.60) | 0.397 | -0.98 (-3.81; 1.84) | 0.492 |
| Tertiary education | 0.22 (-3.28–3.72) | 0.900 | 0.05 (-3.57; 3.68) | 0.976 |
| Employment status |  |  |  |  |
| Unpaid worker/student | Ref | *0.651** | Ref | *0.235** |
| Employed/ self-employed | -1.04 (-3.73; 1.64) | 0.444 | -1.85 (-4.67; 0.97) | 0.197 |
| Employed with high responsibility | -2.19 (-7.59; 3.22) | 0.425 | -4.57 (-10.36; 1.22) | 0.121 |
| Parity |  |  |  |  |
| Nulliparous | Ref | *0.230** | Ref | *0.533** |
| 1–5 | -6.61 (-15.27; 2.06) | 0.134 | -4.70 (-13.42; 4.02) | 0.288 |
| >5 | -7.58 (-16.50;1.34) | 0.095 | -5.07 (-14.02; 3.87) | 0.263 |

**: p-value for the global association with the dependent variable*
